# Excitatory and Feed-Forward Inhibitory Hippocampal Synapses Work Synergistically as an Adaptive Filter of Natural Spike Trains

**DOI:** 10.1371/journal.pbio.0040207

**Published:** 2006-06-20

**Authors:** Vitaly A Klyachko, Charles F Stevens

**Affiliations:** **1**Howard Hughes Medical Institute and Molecular Neurobiology Laboratory, The Salk Institute, La Jolla, California, United States of America; Hebrew UniversityIsrael

## Abstract

Short-term synaptic plasticity (STP) is an important mechanism for modifying neural circuits during computation. Although STP is much studied, its role in the processing of complex natural spike patterns is unknown. Here we analyze the responses of excitatory and inhibitory hippocampal synapses to natural spike trains at near-physiological temperatures. Our results show that excitatory and inhibitory synapses express complementary sets of STP components that selectively change synaptic strength during epochs of high-frequency discharge associated with hippocampal place fields. In both types of synapses, synaptic strength rapidly alternates between a near-constant level during low activity and another near-constant, but elevated (for excitatory synapses) or reduced (for inhibitory synapses) level during high-frequency epochs. These history-dependent changes in synaptic strength are largely independent of the particular temporal pattern within the discharges, and occur concomitantly in the two types of synapses. When excitatory and feed-forward inhibitory synapses are co-activated within the hippocampal feed-forward circuit unit, the net effect of their complementary STP is an additional increase in the gain of excitatory synapses during high-frequency discharges via selective disinhibition. Thus, excitatory and feed-forward inhibitory hippocampal synapses in vitro act synergistically as an adaptive filter that operates in a switch-like manner and is selective for high-frequency epochs.

## Abstract 2

Excitatory and inhibitory hippocampal synapses express complementary short term plasticity components that cooperate to amplify excitatory transmission in response to naturalistic stimulation.

## Introduction

Synapses, commonly considered as computational units of the brain, process information encoded in the spike sequences via a dynamic, history-dependent modulation of synaptic transmission, known as short-term plasticity (STP) [ 1, 2]. Both excitatory and inhibitory synapses express various forms of STP—facilitation, depression, or some mixture of both, depending on the particular type of synapse [ 3– 5], the identity of pre- and/or postsynaptic cells [ 6– 10], and temporal characteristics of the electrical input [ 11– 14]. The interplay of various STP components at each synapse and the dynamic interaction of the two types of synapses are thought to determine the types of computations performed by neural circuits during normal use [ 11– 18].

Several different roles for STP have been proposed in various systems [ 12, 19– 22], including its possible function as a filter for information encoded in spike trains [ 2, 14, 23]. Because of the complex structure of natural spike trains, however, it remains unknown how this filter operates in central synapses and what specific types of information it might select for. Even less understood is the role of interactions between excitatory and inhibitory synapses in the processing of this information. Here we attempted to address these questions using well-defined and easily accessible hippocampal circuitry as a model system.

In the hippocampus, ensembles of cells in the CA3 and CA1 regions produce high-frequency spike discharges when the animal passes through specific locations in the environment (the place fields), thereby providing information about the animal's spatial position and experience [ 24]. During spatial navigation, individual hippocampal neurons alternate between rather long periods of near silence (when the animal is not in that cell's place field) and shorter periods of rapid firing denoting the place field. This general firing pattern is typical of all hippocampal place cells, whereas more specific details, including frequency and duration of discharges, vary widely among cells within each hippocampal region as well as between ensembles of cells in CA1 and CA3 [ 25] and even within single cells over the individual passes through the cell's place field [ 26]. In this study, we used long segments of such spike trains generated by hippocampal place cells in behaving animals [ 26] as a source of spike patterns that hippocampal synapses may encounter in vivo. Below, we refer to these spike trains as “natural” to indicate their origin. Although hippocampal circuit function is not sufficiently well understood to know what characteristics of the spike trains are functionally important, our idea is that this form of stimulation provides synapses with a mixture of interspike intervals, discharge durations, and other statistical properties of stimulus trains that are typical of the ones that synapses might experience naturally in the circuit. Our hope in using this type of stimulation is that we would be able to identify which characteristics of the spike train the STP in hippocampal synapses is tuned to select.

In addition to exciting principal CA1 pyramidal cells, outputs from the CA3 via the Schaffer collaterals branch to activate inhibitory interneurons that provide a feed-forward inhibition onto the same CA1 pyramidal cells [ 27]. Several lines of evidence indicate that feed-forward inhibition is “locked-in” with the excitatory input from the CA3, so that feed-forward inhibitory synapses are reliably activated within a few milliseconds following the excitatory ones. This conclusion is based on the findings that (1) interneurons are reliably driven to fire by the input from pyramidal cells [ 28– 31]; (2) the weakest stimulation of Schaffer collaterals, sufficient to produce a detectable excitatory postsynaptic current (EPSC) in CA1 pyramidal cells, also evoked a delayed inhibitory postsynaptic current (IPSC) [ 32], and (3) focal stimulation of CA3 pyramidal cells resulted in a canonical EPSC–IPSC sequence in the CA1 cells [ 32]. Such a “lock-in” property of feed-forward inhibition, also recently observed in the lateral geniculate nucleus (LGN) [ 33], means that spike trains from the CA3 not only activate excitatory synapses in the CA1, but are also faithfully transmitted by interneurons to rapidly activate feed-forward inhibitory synapses formed onto the same pyramidal cell. This property of feed-forward inhibition provides an opportunity to study, within a functional feed-forward circuit, the role of interactions between excitatory and inhibitory synapses in the processing of natural spike trains.

Several previous studies that used these natural stimulation patterns to investigate STP function in hippocampal CA3-CA1 synapses have shown that, at room temperature, excitatory synaptic responses consist of a complex mixture of facilitation and depression and do not depend on the stimulation pattern alone, because responses differ significantly and in an unpredictable way between different slices and animals [ 13, 18]. Recent studies in hippocampal and cortical synapses have shown, however, that temperature may significantly affect many aspects of synaptic function on both physiological and structural levels [ 34– 38]. We have previously examined the effects of temperature on STP function and found that all components of STP exhibit a strong, but differential, temperature dependence [ 58]. The resulting shift in the expression of the STP components during both constant frequency and natural stimulation has suggested that STP function might be different at room and near body temperatures [ 58].

Here we analyze and compare the responses of excitatory and feed-forward inhibitory hippocampal synapses to natural spike patterns at near-physiological temperatures. We find that excitatory and inhibitory synapses express conserved and complementary sets of STP components that are selective for the epochs of high-frequency discharge associated with hippocampal place fields. Excitatory synapses rapidly increase their gain to a near-constant value during high-frequency epochs, whereas the gain of inhibitory synapses is simultaneously decreased. Unexpectedly, these alterations in gain are mostly independent of the details of the firing pattern during discharges. When excitatory and inhibitory synapses are co-activated within a hippocampal feed-forward circuit unit, the synergistic action of increased excitation and depressed feed-forward inhibition during high-frequency epochs suggests a novel functional role of STP in hippocampal synapses as an adaptive filter selective for high-frequency spike discharges.

## Results

**Figure 1:**
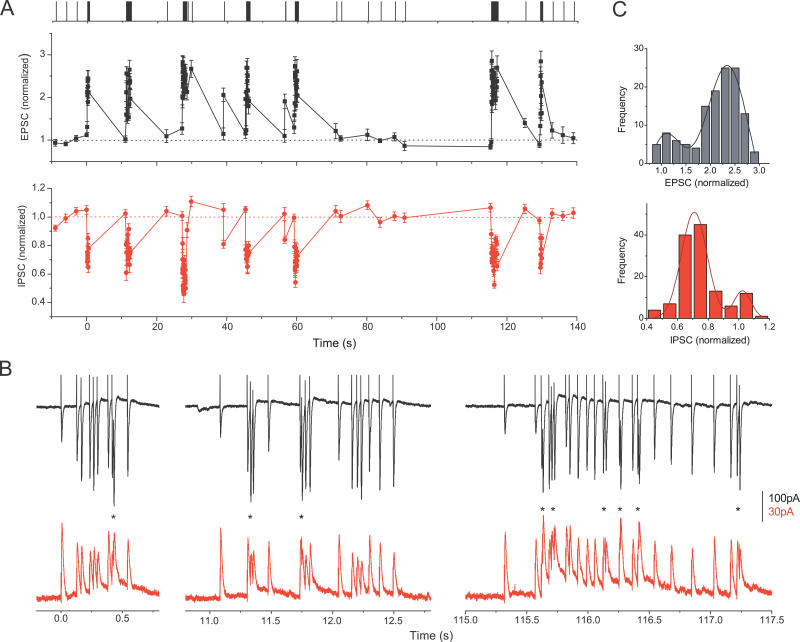
Excitatory and Inhibitory Synapses Act as Adaptive Filters during Natural Spike Trains (A) The top trace illustrates a natural spike train, with each vertical line indicating an action potential in the presynaptic neuron. Relative changes in whole-cell EPSCs (middle trace; black) and IPSCs (lower trace; red) recorded from two different cells during the natural stimulation pattern shown on the top. Each point is an average of four (EPSCs) or seven (IPSCs) recordings from the same cell. Current peak values are normalized to average control values recorded at 0.1 Hz before each stimulation epoch. Four controls before and three after the train are shown, but they are plotted on a shorter timescale for clarity. (B) Three representative raw EPSC (top, black) and IPSC (bottom, red) responses (synaptic current as a function of time) to high-frequency epochs, recorded from the same cells as in (A). Responses to the first, second and sixth epochs are shown. An asterisk (*) denotes frequent cases of summation of closely spaced responses, that, in the case of IPSCs, may occasionally reach levels larger than control (see text for details). (C) Distributions of amplitude changes for data in (A). The amplitude distributions were approximated with two overlapping Gaussians centered at the peaks. Top panel (black) for EPSCs and bottom panel (red) for IPSCs.

Here we used as stimulation patterns the spike trains derived from place-cell firing in freely moving rodents [ 26]. These stimulation patterns consist of short periods of high-frequency discharge (with mean frequency of 28.6 ± 1.2 Hz [4.5–62 Hz] and duration of 12.6 ± 0.9 spikes [3–30 spikes], data from 69 epochs) separated by long periods of low activity ( Figure 1A, top trace). To study the average response of hippocampal synapses to these patterns of stimulation, we used extracellular stimulation of Schaffer collaterals to activate a subset of excitatory fibers that provide input to CA1 pyramidal cells. Relatively weak stimulation intensities were used (average control EPSC amplitude was approximately 5% of maximal) as a compromise between the ability to detect both facilitation and depression and an attempt to replicate more closely the physiologically relevant situation in which CA1 pyramidal cells receive only a small number of synchronous inputs from CA3 pyramidal cells [ 39, 40].

### STP in Both Excitatory and Inhibitory Synapses Acts as an Adaptive Filter Selective for High-Frequency Discharges

Previous recordings performed at room temperature [ 13, 18, 58] have shown that excitatory synaptic responses to natural patterns of stimulation consisted of a complex mixture of facilitation/augmentation and depression that varied significantly among different cells, slices, and animals (the amount of depressed responses varied among different animals from 9% to 70% [ 18, 58]).

At warmer temperatures (33–34 °C, see Materials and Methods for details), natural stimulus trains produced a dramatically different excitatory synaptic response with a highly characteristic pattern ( Figure 1A and 1B, black traces). As at room temperature, excitatory responses underwent large and rapid changes in amplitude (near 3-fold, from 0.87 ± 0.03 to 2.80 ± 0.15 of control, *n* = 36 cells, five patterns). Instead of the complex mixture of facilitation/augmentation and depression, however, excitatory responses rapidly increased during place-field epochs to saturating levels (2.09 ± 0.09 times, [1.37–3.06], *n* = 36), and returned to the baseline during periods of low activity ( Figure 1A, black trace). No apparent depression was observed at any time during natural spike trains since all responses below the unity line were not significantly different from control ( *p* > 0.16 for all).

**Figure 2:**
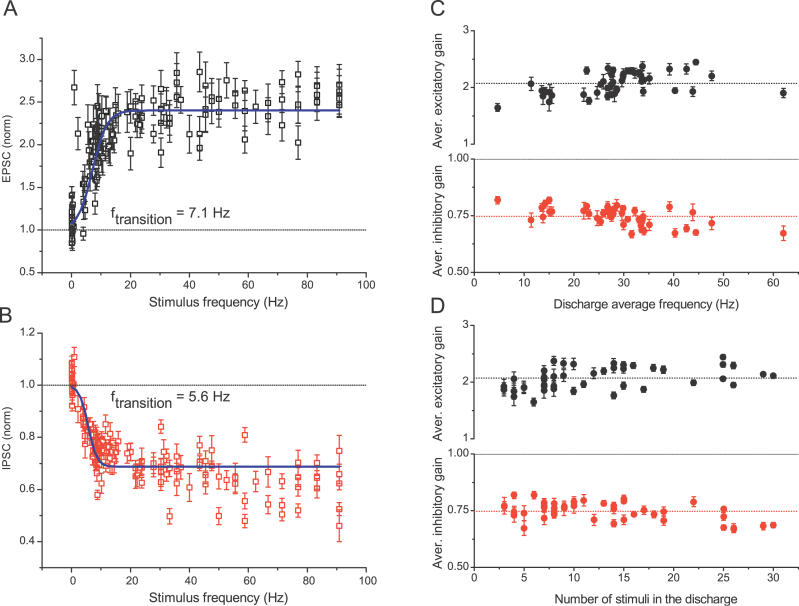
Changes in Excitatory and Inhibitory Synaptic Strength Occur between Two Near-Constant Levels and Are Largely Independent of the Discharge Temporal Pattern (A and B) Excitatory (A) and inhibitory (B) gain values (from Figure 1A) are plotted as a function of the stimulus frequency in the train (1/ISI) and show a rapid transition from one gain level to another. The solid lines represent sigmoidal Boltzmann equation fits with A1 set to 1 and give the transition frequency of 7.1 and 5.6 Hz for excitatory and inhibitory gain changes, respectively. Note that as long as the stimulus is within the discharge, it is likely to result in an elevated gain level, even though its frequency might be below the gain transition range. Two such responses at approximately 1 and 2.5 Hz can be seen in (A), but are infrequent. (C and D) Average changes in excitatory (top) and inhibitory (bottom) synaptic gain during each high-frequency epoch (see Materials and Methods for definition) are plotted as a function of the average stimulus frequency within the epoch (C) and the number of stimuli in the epoch (D) for 44 epochs from five different patterns ( *n* = 36, 14 cells). Note that the plots for excitatory and inhibitory gain changes are shown on different scales.

The distribution of normalized synaptic current amplitudes evoked during the train had only two peaks ( Figure 1C, top): one at 1.10 ± 0.04, corresponding to the inter-epoch EPSC amplitude and the second at 2.09 ± 0.09, corresponding to the increased responses during place-field epochs. The amplitude distribution could be approximated well with two overlapping Gaussians centered at the two peaks ( Figure 1C, top). This two-peak structure of EPSC amplitude distribution was not due to the gaps in the stimulus frequency distribution of the input train ( Figure S1). These results indicate that, at near-physiological temperatures, the excitatory synaptic strength alternates between a near-constant value at low levels of stimulation between high-frequency epochs and another near-constant, but elevated, value during the epochs. In this sense, the excitatory synaptic gain alternates in a switch-like manner. The rapid switching from one gain level to another can be seen more clearly when synaptic response amplitudes are plotted as a function of stimulus frequency ( Figure 2A), and reveal a transition between the levels that occurs at approximately 7 Hz. Rapid changes of synaptic strength between the two levels are also apparent when EPSC amplitudes are plotted as a function of stimulus number rather than time, so that closely spaced responses during discharges can be easily distinguished (several such plots are presented below, see for example Figures 4A, 6B, and 7A). These plots further indicate that the values of synaptic gain during discharges are narrowly distributed around a constant elevated gain level (with a standard deviation of less than ˜15% of the mean).

**Figure 3:**
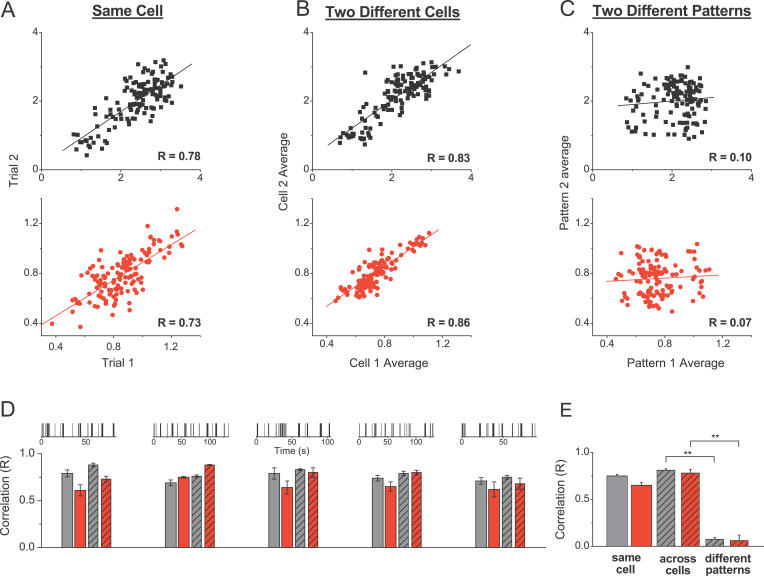
Filtering Patterns of Excitatory and Inhibitory Synapses Are Highly Conserved (A) Normalized response amplitudes during two presentations of the same natural spike pattern (as in Figure 1) for one cell are plotted point-by-point against each other; the correlation coefficient is determined with linear regression. Top panel (black) is for EPSCs and bottom panel (red) for IPSCs. (B and C) Same as (A), but for the average responses from two different cells to the same (B) or to two different (C) stimulation patterns. (D) Top are representations of five different natural spike trains used. Bar graphs below give average correlation values determined as shown in (A–C) for the five different spike patterns. For each pattern, correlations between two presentations for one cell (two left bars, *n* = 4–11 cells/pattern) and between average responses from two different cells (two right bars, *n* = 6–11 random cell pairs/pattern) are plotted for excitatory (black) and inhibitory (red) currents respectively. (E) Summary of the data from (D). ** indicates *p* < 10 ^−6^.

**Figure 4:**
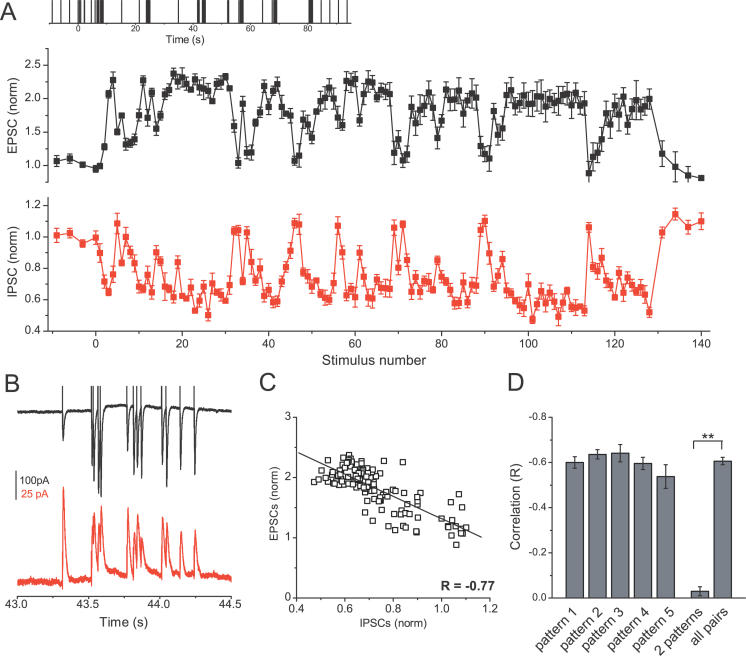
Gain Changes in Excitatory and Inhibitory Synapses Are Simultaneous and (Anti)Correlated (A) Excitatory (top, black) and inhibitory (bottom, red) normalized response amplitudes for a natural spike train (different than that shown in Figure 1) are plotted versus stimulus number within the train. Each point is an average of four (EPSCs) or seven (IPSCs) recordings from the same cells. Four control responses, before and after the spike train, are obtained by stimulation at a constant rate of 0.1 Hz. (B) Representative raw EPSC (top, black) and IPSC (bottom, red) responses (synaptic current as a function of time) to a high-frequency epoch, recorded from the same cells as in (A). (C) IPSC and EPSC amplitudes during the train from (A) are plotted versus each other and the correlation is determined as described in Figures 3A– 3C. (D) Average correlation between changes in EPSC and IPSC amplitudes for five different spike patterns. Ten random pairs of cells are selected for each pattern. ** indicates *p* < 10 ^−18^.

Examination of the data further shows that not only are the values of synaptic gain nearly the same for all stimuli within the discharge, but they are also very similar for all high-frequency epochs in the stimulus train. This point is illustrated in Figure 2, which shows the average excitatory synaptic gain during place-field epochs as a function of the discharge average frequency ( Figure 2C, top) and the number of stimuli in the discharge ( Figure 2D, top). Taken together, the above observations indicate, first, that excitatory synapses recognize and extract specific patterns in the input train associated with place-field discharges and thus operate like a temporal high-pass filter [ 41]. To emphasize that underlying changes in synaptic strength are history-dependent, below we will refer to this synaptic property as an adaptive filtering [ 42]. Second, our data suggest that this filter operates in a switch-like manner, since synaptic strength rapidly alternates between two near-constant levels and is largely independent of the temporal pattern and number of spikes within the discharges. The physiological significance of this filtering pattern is emphasized by the finding that it is highly conserved under a variety of physiological conditions, including a wide range of calcium concentrations (0.7–2 mM) [ 58].

Schaffer collaterals not only excite CA1 pyramidal cells, but also activate inhibitory interneurons that provide feed-forward inhibition onto the same CA1 pyramidal cells. Recent studies have shown that feed-forward inhibition is “locked-in” with the excitatory input, so that activation of feed-forward inhibitory synapses faithfully follows excitation within a few milliseconds [ 32, 33]. Here we took advantage of this property of feed-forward inhibition to study how the interplay between excitatory and feed-forward inhibitory synapses may affect processing of the natural spike patterns in the CA1 pyramidal cells.

Because the common input from the CA3 activates feed-forward inhibitory synapses almost as soon as the excitatory ones, we examined the response of inhibitory synapses to the same input patterns that directly excited CA1 pyramidal cells. This does not imply that pyramidal cells and interneurons have the same firing patterns. Rather, by considering only the correlated firing of pyramidal cells and corresponding feed-forward interneurons, we select for one particular interaction in the hippocampal circuit that occurs during co-activation of these cells by their common input from the CA3 (see later sections for physiological relevance of this approach). In these experiments we blocked excitatory transmission with CNQX or DNQX (10 μM) and applied natural spike trains directly to interneuron cell bodies and local inhibitory arbors in the stratum radiatum. In contrast to the increase in amplitude seen for excitatory currents, inhibitory synapses rapidly depressed during high-frequency epochs to saturating levels (0.73 ± 0.05 of control, [0.65–0.91], *n* = 14 cells, one to five patterns/cell), and returned to control levels between epochs ( Figure 1A and 1B, red traces). Note that overlapping currents often summate to reach levels larger than control ( Figure 1B). Because it is the size of individual responses, but not their summation in the postsynaptic cell, that reflects properties of the synapse, contributions from overlapping synaptic responses were not taken into account in these experiments (see Materials and Methods; summation of synaptic currents/potentials will be discussed below).

The distribution of normalized IPSC amplitudes exhibited just two peaks, one at 1.025 ± 0.023 and another at 0.73 ± 0.05 ( Figure 1C, bottom), corresponding to inter-epoch IPSCs and IPSCs depressed during high-frequency epochs, respectively. Similar to the excitatory synapses, the gain of the inhibitory synapses alternated rapidly between a near-constant value during periods of low activity and another nearly constant, but reduced value during discharges ( Figure 2B). Furthermore, during discharges, the inhibitory synaptic gain was also largely independent of the temporal pattern and number of stimuli in the discharge ( Figure 2C and 2D). Therefore, both types of synapses act as adaptive filters that selectively switch their gain during periods of high-frequency discharge and these history-depended changes in synaptic strength are largely independent of the details of the temporal structure and number of spikes within the discharge.

Studies employing natural spike trains [ 13, 16, 18], including the current one, are forced to discard the fastest inter-spike intervals (ISIs) because the delay between action potential firing and the peak of the postsynaptic response, 3–10 ms for EPSCs/field postsynaptic potentials (fPSPs)/mono- and disynaptic IPSCs, prevents resolution of individual responses at shorter intervals. Can such omission affect the selectivity for high-frequency epochs and their processing, as reported here? We compared synaptic responses to the same natural spike pattern with or without ISIs < 10 ms ( Figure S2) and found that the omission of this very high-frequency component of ISIs did not significantly change the pattern of synaptic response to natural stimulus trains. This result presumably reflects the insensitivity of the synaptic gain changes during a place-field epoch to the precise temporal pattern and number of spikes during the discharge.

### Filtering Patterns of Excitatory and Inhibitory Synapses Are Highly Conserved and Correlated

Since the patterns of responses to a natural spike train at room temperature were not very reproducible across different slices and animals [ 13, 18], we examined reproducibility at near-physiological temperatures. To evaluate reproducibility of responses within a cell, we plotted the normalized amplitudes of responses to each stimulus in the train for one presentation of the train as a function of the corresponding amplitudes during a subsequent presentation of the same train. Reproducibility across different cells was evaluated in the same way, but response amplitudes were first averaged, for each stimulus in the train, across several presentations of the same train within each cell. The correlation coefficient *(R)* in each plot was determined with linear regression. High reproducibility corresponds to a linear plot with a correlation coefficient *(R)* close to 1. We analyzed responses to five different spike patterns of 128 stimuli ( Figure 3), each applied to 4–11 different cells ( *n* = 36 cells for EPSCs and *n* = 14 cells for IPSCs). At near-physiological temperatures, both excitatory and inhibitory responses were highly reproducible not only for a single cell ( *R* = 0.75 ± 0.02 for EPSCs and *R* = 0.65 ± 0.03 for IPSCs) ( Figure 3A), but also across cells from different slices and animals ( *R* = 0.81 ± 0.02 for EPSCs and *R* = 0.77 ± 0.03 for IPSCs) ( Figure 3B and 3E). This high degree of correlation was independent of the particular natural spike train pattern ( Figure 3D). To rule out the possibility that the correlation of responses was an artifact of the spike-pattern structure, we also plotted average responses to two different patterns in 22 pairs of randomly selected cells (11 for EPSCs and 11 for IPSCs) ( Figure 3C). Responses to different patterns were not correlated ( *R* = 0.06 ± 0.06 for EPSCs, *R* = 0.07 ± 0.02 for IPSCs, *p* < 10 ^−6^, compared with the correlation between presentations of the same pattern) ( Figure 3E). These experiments indicate that the selectivity of filtering produced by excitatory and inhibitory synapses is highly reproducible and conserved among different cells and animals.

In addition to sharing a general, but reciprocal, profile of response amplitudes to natural spike patterns, changes in excitatory and inhibitory currents seem to be strongly (anti)correlated. Figure 4A and 4B shows an example of excitatory and inhibitory responses to a different natural stimulation pattern than the one shown in Figure 1, plotted versus stimulus number rather than time to facilitate the comparison of concurrent changes in EPSC and IPSC amplitudes. Again, both excitatory and inhibitory responses follow the same general pattern of increased gain for EPSCs and concomitant decreased gain for IPSCs during high-frequency epochs, and these changes have almost an exact mirror relationship ( Figure 4A and 4B). Correlation analysis performed as described above for Figure 3, confirmed a high degree of correspondence between concomitant changes in EPSC and IPSC amplitudes ( Figure 4C and 4D, *R* = −0.61 ± 0.02, 50 randomly selected pairs, *n* = 50 cells, five patterns). The strong correlation was similar for all five natural patterns tested ( Figure 4D) and was significantly higher than that of non-correlated EPSC/IPSC response pairs, that were associated with two different spike patterns ( *R* = −0.03 ± 0.02, *p* < 10 ^−18^). Taken together, these data suggest that when excitatory and feed-forward inhibitory synapses are co-activated nearly simultaneously by a natural spike train from the CA3, they exhibit highly correlated and conserved responses, aimed at increasing excitation and decreasing inhibition selectively during the periods of high-frequency firing.

### Complementary Sets of STP Components Underlie Reciprocal Filtering Patterns of Excitatory and Inhibitory Synapses

**Figure 5:**
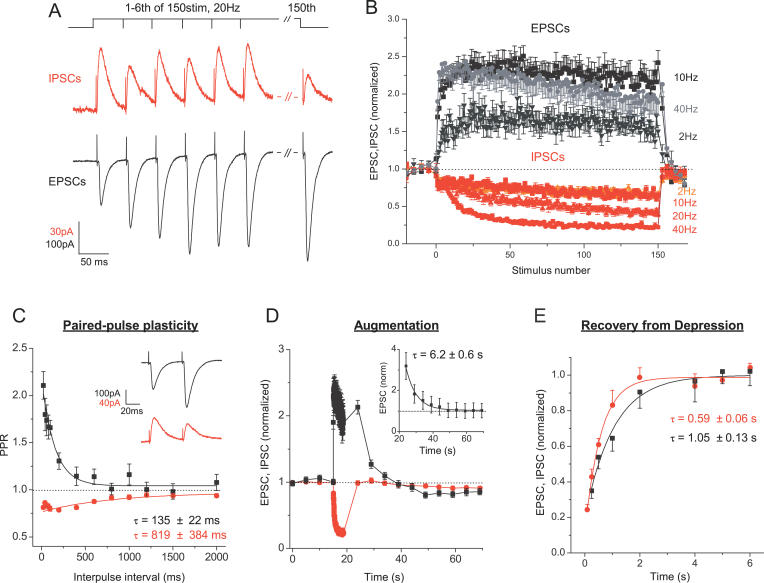
Complementary Expression of STP Components Underlies Opposite Gain Changes of Excitatory and Inhibitory Synapses (A) Top is a representation of the spike train used. Below are whole-cell recordings (current as a function of time) of IPSCs (middle, red) and EPSCs (bottom, black) from two different cells in response to a 150-stimulus, 20 Hz train. First six and 150th responses are shown. (B) Average excitatory (upward going, black) and inhibitory (downward going, red) response amplitudes for 150-stimulus trains at 2, 10, 20, and 40 Hz. Average EPSC response at 20 Hz falls between those at 10 Hz and 40 Hz and is omitted for clarity. Each point is an average of *n* = 9–17 cells for EPSCs and *n* = 5–9 cells for IPSCs. (C) Paired-pulse plasticity, measured at intervals 20 ms to 2 s, for excitatory (black) and inhibitory (red) synapses. Inset. Representative excitatory (top, black) and inhibitory (bottom, red) paired responses (current as a function of time) at a 50-ms interval. (D) A mixture of augmentation and the very slow component of depression is unmasked 5 s after the end of 150 stimuli at 40 Hz. Augmentation was extracted from the mixture using the average monoexponential fit parameters of the very slow depression (τ = 49 ± 9 s, *n* = 13) and assuming a multiplicative relationship between these components [ 44, 45, 58] (inset). (E) Rapid component of depression was studied with the test pulses applied at different intervals (250 ms to 6 s) after 150 stimuli at 40 Hz for excitatory (black) and inhibitory (red) synapses. For EPSCs, recovery from depression was contaminated mostly by the decaying facilitation and augmentation. Rapid component of depression was isolated from the mixture using the analysis introduced by Magleby and Zengel [ 44] and further developed for the hippocampal synapses [ 58], assuming multiplicative relationship among the components and using monoexponential fit parameters of facilitation and augmentation determined for each cell as described in (C) and (D).

What is the mechanism of these selective and reciprocal changes? Previous studies indicate that the distinct forms of STP observed at different types of excitatory synapses are determined by the variable expression of several STP processes [ 11, 14]. Therefore we examined whether differential expression of STP components underlie the reciprocal filtering patterns of excitatory and inhibitory synapses. To test this idea, we used simpler, constant-frequency trains to dissect contributions of different STP components to synaptic modulation. As with natural stimulation patterns, excitatory and inhibitory currents exhibited opposite responses to constant-frequency trains ( Figure 5A and 5B). In the relevant frequency range of 2–40 Hz, EPSCs underwent rapid increases that became larger with frequency and then saturated (increase range: from 1.64 ± 0.08 at 2 Hz to 2.43 ± 0.18 times at 40 Hz, 150-stimulus train, *n* = 14 and 17, respectively) ( Figure 5A and 5B); whereas IPSCs underwent concomitant depression that also became more pronounced with frequency (from 0.68 ± 0.01 at 2 Hz to 0.19 ± 0.03 at 40 Hz, *n* = 6 and 8, respectively).

**Figure 6:**
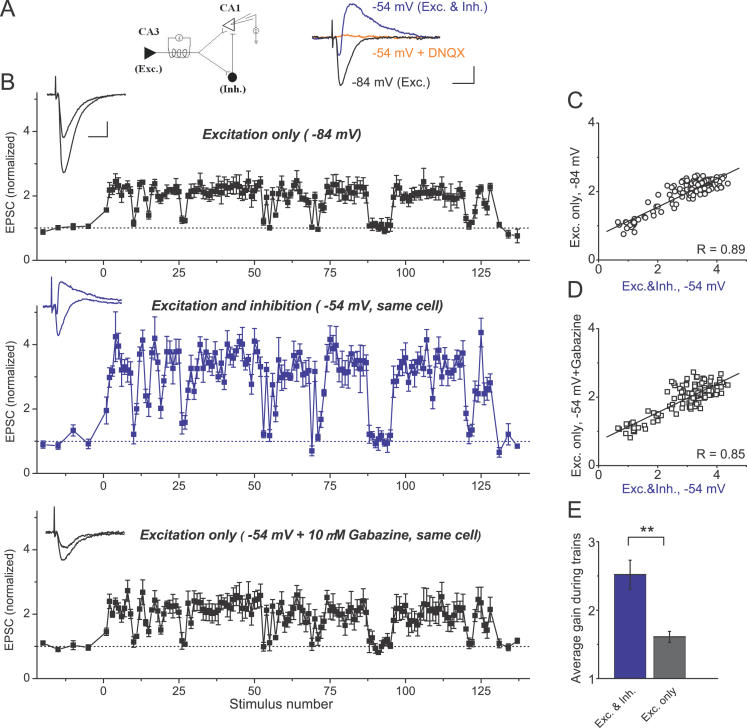
Feed-Forward Inhibition Provides Additional Gain for Excitatory Transmission Selectively during High-Frequency Epochs (A) A schematic of a basic disynaptic feed-forward circuit unit in the hippocampal CA3-CA1 areas (left panel). Pure excitatory responses (Exc.) were isolated by holding the cell membrane near the Cl ^−^ reversal potential (−84 mV) in the absence of AMPA/GABA _A_ receptor antagonists (black sample whole-cell recording trace in the right panel). EPSC/IPSC sequence (Exc. & Inh.) was recorded in the same cell by changing the holding potential to −54 mV (blue sample trace in the right panel). EPSC/IPSC sequence was completely blocked by the AMPA receptor antagonist DNQX (orange sample trace in the right panel), demonstrating the absence of direct stimulation of local inhibitory fibers. Scale bars indicate 10 ms and 100 pA. (B) Top panel: pure excitatory responses to natural stimulus train (same train as in Figure 1) recorded at −84 mV from a different cell than that in (A). The average gain during the train was 1.61 ± 0.08, *n* = 9. The average gain during natural stimulation was significantly larger at −54 mV (middle panel), where excitation and inhibition were present together ( *p* < 0.005, *n* = 9). This additional gain increase was completely blocked at −54 mV by GABA _A_ receptor antagonist gabazine ( *p* > 0.9, bottom panel). Note that in none of these recordings did we subtract contributions from the preceding currents. All recordings shown were from the same cell, and each point was an average of four responses. Each dataset was normalized to its own control. Voltage measurements were a posteriori corrected for the liquid junction potential (see Materials and Methods). Insets: Representative single traces of control current and current with increased gain during one of the discharges are shown for each panel. Scale bars indicate 10 ms and 100 pA. (C) Correlation of response amplitudes from (B) recorded at −84 mV (Excitation only) and at −54 mV (Excitation + Inhibition). (D) Correlation of response amplitudes from (B) recorded at −54 mV after gabazine application (Excitation only) and before gabazine application (Excitation + Inhibition). (E) Average gain during natural spike trains (all points included) for excitation alone (Exc. only) or in the presence of inhibition (Exc. & Inh.); *n* = 9 cells, two different patterns. ** indicates *p* < 0.005.

**Figure 7:**
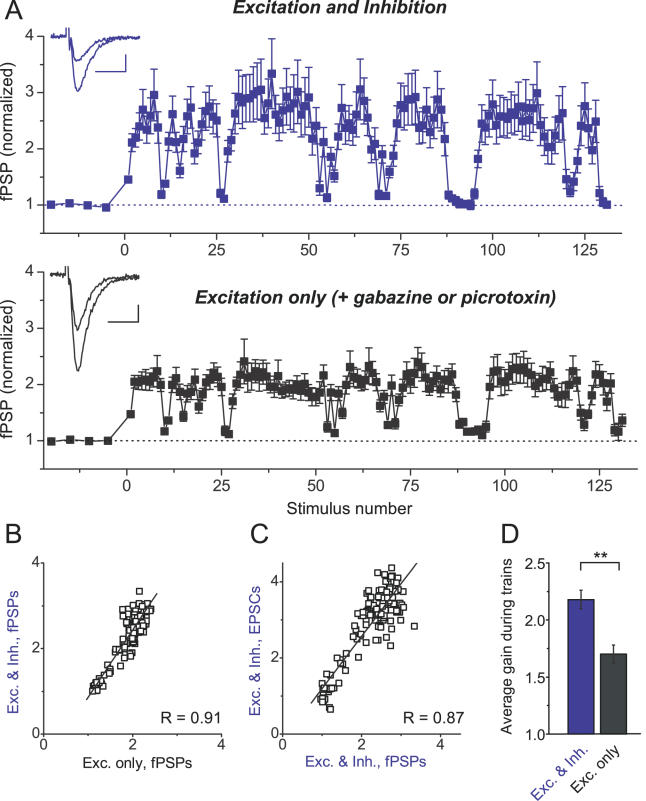
Synergistic Action of Excitation and Feed-Forward Inhibition Is Observed in the Intact Hippocampal Feed-Forward Circuit Unit (A) fPSPs were recorded in hippocampal slices in response to the same natural spike pattern as in Figure 6, in the presence of inhibition (top) or with inhibition blocked by gabazine or picrotoxin (bottom). All recordings shown were from the same slice, and each point was an average of four responses. Insets: Representative single traces of control fPSP and fPSP with increased gain during one of the discharges are shown for each panel. The downward part of the stimulus artifact was removed for clarity. Scale bars indicate 10 ms and 0.3 mV. (B) Correlation of fPSP responses from (A) recorded before and after gabazine application (Excitation + Inhibition vs. Excitation only). (C) Correlation of fPSP and EPSC responses recorded in the presence of inhibition. (D) Average gain of fPSPs (all points included) during natural spike trains for excitation alone (Exc.only) or in the presence of inhibition (Exc. & Inh.); *n* = 7 slices, four different patterns. ** indicates *p* < 0.01.

To investigate further the mechanisms that contribute to these effects, we first isolated contributions from the faster forms of STP by using paired-pulse stimulation insufficient to evoke the slower STP components. Pairs of test pulses separated by 20 ms to 2 s revealed strong paired-pulse facilitation of excitatory currents (1.61 ± 0.17 at 60 ms, *n* = 11) ( Figure 5C), but paired-pulse depression of inhibitory currents (0.83 ± 0.02 at 60 ms, *n* = 9). In addition to rapidly decaying facilitation and depression, longer-term modifications of synaptic strength, augmentation, and the very slow component of depression are known [ 1, 2]. The very slow component of depression [ 43] (τ _decay_ = 49 ± 9 s, *n* = 13, unpublished data) was observed only in excitatory synapses ( Figure 5D) and, at the stimulation levels relevant to the natural spike trains used in this work, the amplitude of this component was negligible (1.02 ± 0.10 at 10 Hz and 0.98 ± 0.06 at 40 Hz, after 50-stimulus train, *n* = 9). To assess augmentation, we recorded EPSCs and IPSCs at 5-s intervals, starting 5 s after the end of 5–150 stimuli, 10–40 Hz trains, when contributions from rapidly decaying components became insignificant ( Figure 5D, examples of the strongest stimulation are shown). Small contributions from slow depression were compensated for as described in [ 44, 45], assuming a multiplicative relationship between the components ( Figure 5D inset; see figure legend and [ 58] for details). Spike trains of all durations and frequencies were sufficient to evoke augmentation of EPSCs (from 1.37 ± 0.06 to 3.19 ± 0.65, 5s after 5- and 150-stimulus trains at 10 and 40 Hz respectively, *n* = 6 and 17, respectively). In contrast, even the strongest stimulation (150 stimuli) failed to evoke augmentation of inhibitory currents at any of the frequencies tested (IPSC amplitude was 0.93 ± 0.05 at 10 Hz and 0.99 ± 0.03 at 40 Hz, 5 s after the train, *n* = 9 and 6, respectively). Thus, the responses of excitatory synapses are dominated by facilitation and augmentation, whereas inhibitory synapses express only depression. This analysis demonstrates that excitatory and inhibitory inputs into CA1 pyramidal cells express complementary sets of STP components that underlie the opposite profile of responses to natural spike patterns.

As shown in Figure 5, we observed a net increase in excitatory responses at all frequencies and stimulus durations relevant to natural spike trains (2–40 Hz, 5–150 spikes). But excitatory hippocampal synapses are known to depress at room temperature during continuous activity as a result of vesicle depletion [ 5, 35]. Therefore, we asked whether depression was absent at higher temperatures or whether it was masked by dominating facilitation and augmentation. Depression was assessed with the test pulses applied at different intervals (250 ms to 6 s) after 150-stimulus train at 40 Hz ( Figure 5E). After correcting synaptic responses for contributions from facilitation and augmentation [ 45] (see also figure legend for details), we found that, at 33–34 °C, EPSCs depressed during the train at least as much as IPSCs (0.35 ± 0.04 vs. 0.43 ± 0.04 at 250 ms after the train, *n* = 9 and 5, respectively) ( Figure 5E). Therefore, although masked by facilitation and augmentation, significant depression accumulates during trains and may act to counterbalance these gain-increasing components of STP during high-frequency discharges (unpublished data).

### Synergistic Action of Excitation and Feed-Forward Inhibition in the Hippocampal Feed-Forward Circuit Unit

What is the physiological significance of the inverse responses to natural stimulation patterns exhibited by excitatory and feed-forward inhibitory synapses onto CA1 pyramidal cells? Our data suggest that in the hippocampal feed-forward circuit unit—when both excitation and “locked-in” feed-forward inhibition are co-activated by a common input from the CA3—selective depression of feed-forward inhibition would provide an additional increase in excitatory synaptic gain during periods of high-frequency firing. To test this idea, we stimulated Schaffer collaterals in the absence of AMPAR or GABA _A_R antagonists, and compared responses to natural spike patterns evoked in CA1 pyramidal cells at two holding potentials ( Figure 6): at −84 mV, the Cl ^−^ reversal potential, where only excitation was present due to null Cl ^−^ driving force, and at −54 mV, where excitation was rapidly followed by feed-forward inhibition in a canonical EPSC–IPSC sequence [ 32] ( Figure 6A, right panel). At −54 mV, both EPSCs and IPSCs were completely blocked by AMPA receptor antagonist DNQX (orange trace in Figure 6A), confirming that inhibitory interneurons were interposed between the Schaffer collaterals and the inhibitory synapses and, therefore, the contribution from the direct stimulation of local inhibitory fibers was negligible. It is also important to note that the experiments described below are not based on the assumption that excitatory and feed-forward inhibitory synapses are co-activated nearly simultaneously as a result of Schaffer collateral stimulation, but rather provide a test for this assumption.

At −84 mV, pure excitatory currents followed exactly the same pattern of response to natural stimulus train, as described above, with a 1.61-fold (± 0.08) average increase in response size during the train ( *n* = 9, all responses included) ( Figure 6B, top). At −54 mV the contribution from feed-forward inhibition became evident as an EPSC was rapidly followed by a large IPSC ( Figure 6B, middle). As expected, the presence of feed-forward inhibition significantly increased the average excitatory gain during the train to 2.52 ± 0.21 ( *p* < 0.005, Figure 6B, middle, and Figure 6E), leaving the overall profile of the response unchanged ( Figure 6C; *R* = 0.74 ± 0.04, *n* = 9). To control for possible differences in excitatory transmission or STP at −84 mV versus −54 mV, we then blocked inhibition with a selective GABA _A_R antagonist, gabazine ( Figure 6B, bottom, and Figure 6D). With inhibition blocked, the increase of excitatory responses at −54 mV was indistinguishable from that at −84 mV (average gain 1.59 ± 0.19, *p* > 0.91, *n* = 9). Therefore, the additional EPSC amplification is mediated solely by the presence of feed-forward inhibition, and this extra amplification can be eliminated by two different methods of blocking inhibitory transmission. Because this action of feed-forward inhibitory synapses was predicted from the direct stimulation of inhibitory fibers, these data provide additional support to the earlier observations that activation of feed-forward inhibition is locked-in with the input from the CA3.

The interpretation of the synergistic action between excitatory and feed-forward inhibitory inputs to CA1 pyramidal cells, as observed here, is complicated by the fact that the relative contribution of inhibitory currents and their summation as reported by whole-cell recordings depends on a combination of the holding potential and the reversal potential of Cl ^−^. This problem remains in whole-cell current-clamp recordings since the relative contribution of inhibition is still determined by the composition of the pipette solution that sets the reversal potential of Cl ^−^. Certain configurations of the perforated patch recordings preserve the natural internal Cl ^−^ concentration, but this technique is prone to significant space clamp problems and is not sufficiently stable for prolonged recordings. We, therefore, sought to confirm these findings in the feed-forward hippocampal circuit by fPSP recordings ( Figure 7). In this case, we record from a population of completely intact neurons so that intracellular ionic composition and cell membrane potential are undisturbed, and the summation of consecutive inputs and the interplay between excitation and feed-forward inhibition remains intact.

The profile of excitatory synaptic responses to the same natural stimulation pattern was remarkably similar in field potential and whole-cell recordings ( Figure 7A and 7C; *R* = 0.82 ± 0.02, *n* = 30 randomly selected pairs). As in whole-cell recordings, the magnitude of the net increase in fPSP responses during natural spike trains was significantly larger when both excitation and inhibition were present (2.18 ± 0.08, Figure 7A, top), compared to that of excitation alone (1.70 ± 0.08, *p* < 0.01, *n* = 7 slices) ( Figure 7A, bottom, and Figure 7D) and, as before, the pattern of the response remained unchanged ( Figure 7B; *R* = 0.81 ± 0.04, *n* = 7). Since blocking inhibition was associated with an increase in fPSP amplitude (73 ± 16%, *n* = 7), the above effect could arise from response saturation rather than excitation-inhibition interactions. To eliminate this possibility, we induced a similar increase in fPSP size (66 ± 8%, *p* > 0.7, *n* = 14) by changing the stimulation intensity. Such an increase also produced a reduction of the average increase in response size during the train by a small (˜10%), but significant amount ( *p* < 0.01). This can account for approximately 1/3 of the effect of blocking the inhibition. Thus, the major effect of feed-forward inhibition is the specific one in which it serves to magnify the already potentiated excitatory potentials during periods of high-frequency discharge via a selective decrease in inhibitory gain.

## Discussion

Our results indicate that (1) at near-physiological temperatures, excitatory and inhibitory hippocampal synapses have precisely tuned sets of STP components that rapidly change synaptic strength specifically during the epochs of high-frequency discharge commonly associated with hippocampal place fields; and (2) due to the expression of complementary STP, excitatory and feed-forward inhibitory synapses cooperate in the selective amplification of excitatory transmission during high-frequency discharges. Surprisingly, synaptic strength at both types of synapses is largely independent of the discharge temporal pattern and alternates between two near-constant levels, one during periods of low activity and another (elevated for excitatory synapses or reduced for inhibitory synapses) during discharges. Taken together, our observations show that excitatory and feed-forward inhibitory synapses, co-activated within the hippocampal feed-forward circuit in vitro, act as an adaptive filter that operates in a switch-like manner and is selective for high-frequency epochs.

By using as stimulation patterns spike trains recorded in the hippocampus of behaving rodents, we attempted to identify characteristics of the naturally occurring spike trains that STP in hippocampal synapses might be selective for. Although the specific details of the spike patterns vary widely among individual cells in each hippocampal subfield and among ensembles of cells in CA3 and CA1 [ 25, 26], the patterns used here provide a range of interspike intervals, discharge frequencies, and durations as well as a general structure characteristic of the inputs that hippocampal synapses would experience naturally. A striking feature of our findings is that the changes in synaptic gain produced by the STP filter are largely independent of the exact details of the temporal structure and number of spikes within the discharges ( Figures 1 and 2).

Epochs of high-frequency discharge are a common firing pattern of pyramidal neurons in many brain areas and especially in the hippocampus, where high-frequency discharges of place cells provide activity-encoded information about spatial position within the environment [ 24]. If epochs of rapid firing are an important feature of a spike train containing specific information, as believed by many investigators (reviewed in [ 46]), then the STP mechanisms exhibited by excitatory and inhibitory synapses, as reported here, provide a precise filtering system to sense and amplify this information. This filter is constructed, for excitatory synapses, by combining facilitation and augmentation that provide selectivity and amplification of high-frequency epochs in a wide temporal range, with masked depression that may act to counterbalance these gain-increasing components of STP, thus limiting potentiation during the epochs (unpublished data). In the case of inhibitory synapses, selectivity and gain control are determined by a much simpler STP paradigm, consisting of a single component—synaptic depression. Although paired-pulse plasticity and other forms of STP at excitatory and inhibitory synapses have been known for decades [ 3– 5, 9, 10], the functional roles of facilitation and augmentation in information processing have remained largely unknown, whereas the role of depression has been established only in a few specific cases, when it dominated the synaptic response [ 12, 19– 22]. While inhibitory synapses fall into the same category, our analysis of excitatory hippocampal synapses suggests possible functional roles for all STP components within a complex STP paradigm.

Despite the different STP mechanisms underling filtering patterns of excitatory and inhibitory synapses, they operate in a similar, highly non-linear manner: Synaptic strength rapidly changes from a near-constant value at low levels of stimulation between high-frequency epochs to another near-constant value during the epochs ( Figures 1 and 2). Surprisingly, our data also show that the value of the synaptic gain during discharges is largely independent of the temporal pattern and number of spikes within the discharge ( Figure 2). Together these observations indicate that, during stimulation with natural spike trains, synaptic strength in both types of synapses alternates in a switch-like manner. Similar to many other biological switches [ 47, 48], the switching of synaptic gain is not perfectly binary: Intermediate gain values are present and changes between the two levels are not immediate, although relatively fast ( Figures 1 and 2). It is interesting to note that in both types of synapses transition between the two gain levels occurs at frequencies around 5–7 Hz, which corresponds to the lower bound of the discharge average frequency in our stimulation patterns. As a result, for nearly all discharge frequencies relevant to natural spike trains used in our study, namely 5–62 Hz, the incoming high-frequency epochs are reliably detected in both excitatory and inhibitory synapses ( Figure 2). Thus the properties of STP seem to be finely tuned to the frequencies in the input train relevant to place-field discharges and allow the detection of the information-carrying epochs in a wide frequency range.

The selectivity of the STP filter for high-frequency epochs was observed, not only at the level of individual synapses ( Figures 1– 4), but also in the hippocampal feed-forward unitary circuit, by stimulating the common input to CA1 pyramidal cells and the inhibitory interneurons that provide feed-forward inhibition onto the same CA1 pyramidal cells ( Figures 6 and 7). In the latter experiments, the presence of feed-forward inhibition produced additional amplification of the excitatory synaptic gain during discharges via selective disinhibition. Such synergistic action was predicted from the direct stimulation of inhibitory and excitatory fibers, assuming that they receive nearly simultaneous input. These results, therefore, support earlier observations that feed-forward inhibition is locked-in with the excitatory input into CA1 [ 32]. A large number of in vitro and in vivo studies support this observation, by demonstrating that synapses formed by the hippocampal pyramidal cells onto inhibitory interneurons are highly reliable [ 28– 32]. Moreover, because pyramidal cells in CA3 are known to fire in ensembles [ 39, 40], interneurons that can be driven to fire by a single EPSP [ 28, 49, 50] are likely to transmit such synchronous inputs faithfully. This locked-in property of feed-forward inhibition provides justification for using the same spike trains as an input to both excitatory and feed-forward inhibitory synapses in order to study their interaction, and might be a general property of feed-forward loops as similar locked-in feed-forward inhibition has been recently reported in the LGN [ 33].

The physiological significance of the filtering paradigm observed in hippocampal synapses at near body temperatures is emphasized by its high reproducibility among different cells, which is in a striking contrast to the large variability of excitatory synaptic responses observed at room temperature [ 13, 18]. What is the mechanism of these temperature-dependent changes? Analysis of the role of temperature in STP function [ 58] reveals large differences in the expression of underlying STP processes with temperature. These differences arise from a strong, but differential, temperature dependence of all major forms of STP. At room temperature, frequency-dependent depression overwhelms weak potentiation during trains, and the ratio of these processes depends on the stimulus frequency and duration and also varies with the animal's age [ 18]. As a result, the relative contributions of potentiation and depression vary among different high-frequency epochs in each cell as well as among different cells. In contrast, at higher temperatures, depression is well balanced by increased facilitation and augmentation [ 58] and, in fact, is masked at any frequency and stimulus duration relevant to the natural spike trains independently of the animal's age. Consequently, responses during high-frequency epochs are always potentiated, and variability among different cells is significantly reduced.

Taken together, our data establish that excitatory transmission in vitro is (1) amplified selectively during high-frequency epochs due to a precisely tuned set of STP components and (2) is aided by feed-forward inhibitory synapses that provide additional amplification during high-frequency discharges via selective disinhibition. Previous studies emphasized the role of inhibition in the dynamic control of hippocampal pyramidal cell output via its involvement in the generation of rate and temporal coding [ 51, 52], setting limits to the integration window for spike generation and coincidence detection [ 17, 32], and by synchronizing pyramidal cell firing [ 53]. The synergistic action of excitatory and feed-forward inhibitory synapses described here is a novel feature of feed-forward circuits that is revealed by the application of natural stimulation patterns at near-physiological temperatures. The amplification of information-carrying epochs via depression of inhibition occurred in response to spike patterns recorded in the exploring animal, and might be relevant to hippocampal function in learning, since inhibition is also known to be depressed in the hippocampus of freely moving rodents during the exploration of a novel environment [ 54].

Since feed-forward loops are recognized as one of the essential elements of the neuronal circuits [ 27], our results provide the foundation to the future understanding of the hippocampal function in vivo. Indeed, in vivo recordings also found a 3-fold gain of excitation over inhibition during periods of high-frequency discharge [ 55]. However, this effect was observed during much faster sharp-wave oscillations and future in vivo recordings during place-field epochs will be needed to determine how the interactions within the feed-forward circuit unit described here are manifested in the more complex in vivo hippocampal circuitry. Because high-frequency discharges of hippocampal pyramidal cells are often associated with place fields, it is tempting to speculate that the filtering mechanisms observed here may potentially be used in vivo to amplify the place-field responses. However, such a projection to the in vivo level cannot be made until the patterns of connectivity that govern the distribution of the place field–related activity between pyramidal cells and interneurons are established. Moreover, one should keep in mind that the interactions between synaptic responses observed here will be further complicated in vivo by several factors, including the presence of feed-back loops, additional inputs to the CA1 pyramidal cells directly from the entorhinal cortex [ 56], the complex and poorly understood connectivity between functional subgroups of interneurons and pyramidal cells, and a resulting summation of multiple inputs from different sources in both types of cells. In addition, in our slice experiments, multiple presynaptic fibers were activated with the same stimulation patterns in order to study the average behavior of multiple parallel feed-forward circuit units with very similar characteristics. In the hippocampal circuitry in vivo, synaptic interactions can be further complicated by the fact that different presynaptic CA3 neurons might show different spiking patterns and the spikes are less precise than those induced here with electrical stimulation.

Nevertheless, the present study represents a step forward in understanding STP function in information processing by individual synapses and during their interaction within the context of a functional feed-forward unitary circuit. Moreover, we believe that the role of STP as an adaptive filter selective for the information-carrying features in the spike train, as suggested by present experiments, may be one of the major STP functions in synaptic computations, and could occur in many brain areas. In this case, patterns of STP expressed by central synapses are likely to vary among different brain regions as it would be selective for the types of information relevant for a particular brain area. This idea is supported by the findings that structurally similar excitatory synapses in the cortex and hippocampus express very different STP paradigms [ 12, 19] as they process information encoded by different natural spike patterns [ 57]. It remains to be seen whether such information-specific tuning of STP operates in other brain regions and which cellular and molecular mechanisms determine information-specific paradigms of STP expression in various brain areas.

## Materials and Methods

### Preparation.

The 400-μm transverse hippocampal slices were prepared from 14–25-d-old Long Evans rats for whole-cell recordings and 21–35-d-old rats for fEPSP recordings, as described elsewhere [ 10, 32]. In brief, slices were cut on a Leica Vibrotome (Leica Microsystems, Wetzlar, Germany) in ice-cold modified ACSF, containing: 85 mM NaCl, 75 sucrose, 2.5 mM KCl, 1.25 mM NaH _2_PO _4_; 26 mM NaHCO _3_; 0.5 mM CaCl _2_; 4 mM MgCl _2_, 13 mM glucose, bubbled with 95% O _2_/5% CO _2_. Slices were incubated for approximately 1 h at 32 °C and then held at room temperature for 0–4 h before recordings in standard ACSF solution, of the same composition as above, but with 125 mM NaCl, 2 mM CaCl _2_, 1 mM MgCl _2_, and no sucrose. The CA3 region was surgically separated in each slice with incision to prevent recurrent excitation.

During experiments, bath temperature in the recording chamber was continuously monitored and adjusted to 33–34 °C with an automatic heater controller. This choice of recording temperature is based on the analysis of STP temperature dependence, indicating that changes in the kinetics and amplitudes of all STP components occur mostly in the 23–33 °C temperature range, but the parameters of STP components are invariant over the temperature range 33–38 °C. Because it is difficult to maintain stable slices for prolonged periods of time at temperatures above 33–34 °C (due to the oxygen depletion of the solution at these temperatures), all recordings were performed at the highest near-physiological temperatures that allowed stable recordings, 33–34 °C. We confirmed, however, that synaptic responses at 33–34 °C did not differ significantly from those at 37–38 °C (see [ 58] and also data in Figure S2 that were recorded at 37–38 °C).

### Electrophysiological recordings.

Schaffer collaterals were stimulated (100 μs, 50–200 μA current injections) via a bipolar electrode placed in the stratum radiatum, and either whole-cell currents or extracellular field potentials were recorded using an Axopatch 200B amplifier (Molecular Devices, Sunnyvale, California, United States). EPSCs were recorded from CA1 pyramidal cells in the presence of the GABA _A_R antagonist picrotoxin (100 μM) or the more-specific GABA _A_R antagonist gabazine (10 μM) at the holding potential of −74 mV, unless noted otherwise. Average control EPSC was 104 ± 5 pA, corresponding to activation of approximately 30 fibers (taking quantal size as ˜10 pA and an average release probability as ˜0.3). Micropipettes (3–6 MΩ) were filled with solution containing: 130 mM K-gluconate, 10 mM KCl, 9 mM NaCl, 10 mM HEPES, 5 mM EGTA, 2.5 mM MgATP, 0.3 mM LiGTP (pH 7.25, 290–300 mOsm). Monosynaptic IPSCs were evoked by stimulating local axonal arbors and cell bodies in the stratum radiatum in the presence of the AMPAR antagonist CNQX or DNQX (10 μM). In preliminary experiments, IPSCs were recorded with the same pipette solution as described above; most of IPSC recordings and simultaneous EPSC/IPSC recordings were made at −49 mV to −59 mV with the modified pipette solution, formulated to increase Cl ^−^ driving force by the following changes: 140 mM KGluconate, 4 mM NaCl, 0 mM KCl, and 15 mM HEPES. For whole-cell and fPSP recordings of excitation/inhibition interactions the stimulus electrode was positioned as far away from the recording site as possible, to minimize contributions from monosynaptic IPSCs. In all experiments NMDA receptors were blocked with 50 μM AP-5 to prevent possible long-term effects. Membrane holding potentials were corrected a posteriori for the experimentally determined liquid junction potential (LJP = 14 ± 3 mV). Access resistance was continuously monitored, and cells with unstable access resistance were discarded from analysis.

### Natural spike patterns.

Natural stimulation patterns used here represent timings of action potential firing recorded in vivo from hippocampal place cells of awake, freely moving rats [ 26]. These spike patterns consist of short periods of high-frequency discharge, defined as three or more spikes with ISIs less than 1 s, separated by long periods of low activity. Five different patterns of 128 stimuli were used. Both constant frequency and natural stimulation patterns were presented multiple times to the same cell or slice, separated by 2–3-min control sections at 0.1 Hz. Each subset of data was normalized to an average of four to six immediately preceding control responses. Because the delay between the action potential firing and the peak of postsynaptic currents/potentials (4–10 ms for EPSCs/fPSPs/monosynaptic and disynaptic IPSCs) prevented resolution of individual synaptic responses at shorter ISIs, we treated spikes with ISI less than 10 ms as a single stimulus [ 13]. Control experiments (see Figure S2) confirmed that the removal of ISIs less than 10 ms did not significantly affect the synaptic response to natural stimulation patterns.

### Data analysis.

Data were analyzed using software locally written in Matlab. Both peak amplitudes and initial slopes were analyzed and compared for consistency. Peak amplitudes were measured relative to the baseline calculated as an average of five points (1 ms) immediately preceding each spike. To account for the overlap of currents or potentials, that occurs at short ISIs, the following method was applied to each set of responses to 128 stimuli presentation and six to eight preceding control measurements: First, a template of EPSC, IPSC, or fPSP waveform was created by averaging responses separated by at least 100 ms (200 ms for IPSCs) from their neighbors and normalized to their peak values. For all ISIs less than 40 ms (80 ms for IPSCs), two previous responses were approximated by a template waveform scaled to their peaks, and the contributions to the current response were subtracted. Because of the time course of EPSC (˜10 ms) and IPSC (˜17 ms) decay and the delay between action potential firing and the peak of the postsynaptic response (4–10 ms), the contribution from the third preceding response during high-frequency epochs was indistinguishable from noise for all types of recordings. This correction was carried out for all recordings except for the experiments with excitation/inhibition interactions in Figures 6 and 7 (see text for details). All data are presented as mean ± standard error of the mean. Statistical significance was evaluated using two-tailed *t-*test.

## Supporting Information

Figure S1 The Two-Peak Structure of EPSC (or IPSC) Amplitude Distribution during Natural Spike Trains Is Not Due to the Gaps in the Stimulus Frequency Distribution of the Input Train (A–C) The stimulus frequency distribution is plotted for the stimulus pattern in Figure 1 (A), for the stimulus pattern in Figure 4 (B), and for all available patterns (C). Insets: In each panel, the stimulus frequency distribution is plotted for the frequency range of 1–10 Hz on an expanded scale.
(1.3 MB EPS)

Figure S2 The Removal of ISIs Less Than 10 ms Does Not Change the Pattern of Synaptic Response to Natural Spike Trains (A and B) A segment of natural stimulation pattern was used that contained two high-frequency epochs flanked by periods of low activity ([B], top). The exact timings of stimuli during the second epoch are shown in (A) on an expanded timescale. Arrows indicate the stimuli with ISI less than 10 ms that were added/removed. Excitatory synaptic responses were evoked at 37–38 °C by the stimulation pattern above that contained all ISIs (blue trace) or when ISIs less than 10 ms were removed (same cell, black trace). Representative synaptic responses to the second epoch are shown in (A) on an expanded timescale, and the average normalized EPSC initial slopes from the same cell are plotted in (B) versus stimulus number (four train presentations each). The presence of ISIs less than 10 ms (or their removal) did not affect the history dependence of synaptic responses and the selectivity for high-frequency discharges. The same results were found in responses to two different natural stimulation patterns in *n* = 6 cells both at 33 °C and 37–38 °C. Note that slopes of synaptic responses had a slightly larger variability when ISIs less than 10 ms were present (CV _no ISIs<10ms_ = 0.24 ± 0.02, CV _all ISIs_ = 0.29 ± 0.02). This likely reflects a larger uncertainty in automated determination of EPSC slopes in cases of strongly overlapping synaptic responses with ISIs less than 10 ms.
(1.1 MB EPS)

## Abbreviations

EPSC: excitatory postsynaptic current
fPSP: field postsynaptic potential
IPSC: inhibitory postsynaptic current
ISI: inter-spike interval
STP: short-term plasticity
